# Uncovering the Novel QTLs and Candidate Genes of Salt Tolerance in Rice with Linkage Mapping, RTM-GWAS, and RNA-seq

**DOI:** 10.1186/s12284-021-00535-3

**Published:** 2021-11-14

**Authors:** Weilong Kong, Chenhao Zhang, Shengcheng Zhang, Yalin Qiang, Yue Zhang, Hua Zhong, Yangsheng Li

**Affiliations:** 1 State Key Laboratory of Hybrid Rice, College of Life Sciences, Wuhan University, Wuhan, 430072 China; 2 Shenzhen Branch, Guangdong Laboratory for Lingnan Modern Agriculture, Genome Analysis Laboratory of the Ministry of Agriculture, Agricultural Genomics Institute at Shenzhen, Chinese Academy of Agricultural Sciences, Shenzhen, 518120 China

**Keywords:** Salt stress, Rice (*Oryza sativa* L.), Linkage mapping, RTM-GWAS, RNA-seq

## Abstract

Salinity is a major abiotic stress that limits plant growth and crop productivity. *Indica* rice and *japonica* rice show significant differences in tolerance to abiotic stress, and it is considered a feasible method to breed progeny with stronger tolerance to abiotic stress by crossing *indica* and *japonica* rice. We herein developed a high-generation recombinant inbred lines (RILs) from Luohui 9 (*indica*) X RPY geng (*japonica*). Based on the high-density bin map of this RILs population, salt tolerance QTLs controlling final survival rates were analyzed by linkage mapping and RTM-GWAS methods. A total of seven QTLs were identified on chromosome 3, 4, 5, 6, and 8. *qST-3.1*, *qST-5.1*, *qST-6.1*, and *qST-6.2* were novel salt tolerance QTLs in this study and their function were functionally verified by comparative analysis of parental genotype RILs. The gene aggregation result of these four new QTLs emphasized that the combination of the four QTL synergistic genotypes can significantly improve the salt stress tolerance of rice. By comparing the transcriptomes of the root tissues of the parents’ seedlings, at 3 days and 7 days after salt treatment, we then achieved fine mapping of QTLs based on differentially expressed genes (DEGs) identification and DEGs annotations, namely, *LOC_Os06g01250* in *qST-6.1*, *LOC_Os06g37300* in *qST-6.2*, *LOC_Os05g14880* in *qST-5.1*. The homologous genes of these candidate genes were involved in abiotic stress tolerance in different plants. These results indicated that *LOC_Os05g14880*, *LOC_Os06g01250*, and *LOC_Os06g37300* were the candidate genes of *qST-5.1*, *qST-6.1,* and *qST-6.2*. Our finding provided novel salt tolerance-related QTLs, candidate genes, and several RILs with better tolerance, which will facilitate breeding for improved salt tolerance of rice varieties and promote the exploration tolerance mechanisms of rice salt stress.

**Supplementary Information:**

The online version contains supplementary material available at 10.1186/s12284-021-00535-3.

## Introduction

Soil salinization seriously affects seed germination, plant growth, and crop production (Yang and Guo 2018a; Liu et al. 2021). According to statistics, saline-alkali land is widely distributed worldwide, with a total area of about one billion hectares, affecting 1/4 to 1/3 of crop production (Munns 2002). In recent years, climate change has increased soil salinization (Kong et al. 2019; Liu et al. 2021). As the most important food crop in the world, rice (*Oryza sativa* L.) provides a source of food for more than half of the world’s population (Kong et al. 2019). However, rice is a moderately salt-sensitive crop, and increasing soil salinization has become an important factor restricting rice production and threatening food security (Ganie et al. 2021). Therefore, it is of great significance to understand the physiological, biochemical and molecular mechanisms of salt tolerance in rice.

In plants, salt stress affects almost all developmental stages from germination to senescence including osmotic and ion stress, and the resulting reactive oxygen species (ROS) stress, and nutrient consumption (Hanana et al. 2011). When the salt content of the soil increases, the water potential of the soil solution will be lower than the water potential of the plant root cells, which results in the inhibition of root water absorption, and plants must perform osmotic adjustment to maintain cell expansion, growth, as well as water absorption (Fricke et al. 2004; Hakim et al. 2014). In addition, osmotic stress can cause stomatal closure, which inhibits the absorption of carbon dioxide by plants and leads to the decrease of photosynthesis (Wegner et al. 2011; Qin and Huang 2020; Zhao et al. 2020). Ion stress is mainly caused by the accumulation of sodium (Na^+^) and chlorine (Cl^−^) in cells (Rana et al. 2008; Yang and Guo 2018b). The toxicity of sodium is mainly that sodium has an inhibitory effect on enzyme activity and negatively affects metabolism including the Calvin cycle and other pathways (Cheeseman 2013; Wu et al. 2018). What’s more, excessive sodium in the cytoplasm will also interfere with the absorption and transportation of potassium and mineral elements such as nitrogen, phosphorus, potassium, calcium, and zinc (Shabala and Pottosin 2014; Munns et al. 2016; Iqbal et al. 2018; Seifikalhor et al. 2019; Razzaq et al. 2020). Since the absorption channels of NO3^−^, SO_4_^2−^, and Cl^−^ are absorbed by the same non-selective anion transporter, excessive Cl^−^ will lead to the lack of key macronutrient nitrogen and sulfur (Rana et al. 2008; Liu et al. 2021). In addition to osmotic and ion stress, salt stress can also lead to the accumulation of ROS in cells, which can severely damage cell structures and macromolecules, such as DNA, lipids, and enzymes (Miller et al. 2010; Ahanger et al. 2017).

Rice salt tolerance is a comprehensive manifestation of a variety of physiological and biochemical reactions and is a quantitative trait controlled by multiple genes and has a complex genetic basis (Kong et al. 2019; Liu et al. 2021). However, due to the complexity of salt tolerance in rice, there are very few QTLs that have been finely mapped or cloned among the numerous salt tolerance QTLs. Lin et al. (2004) detected a major QTL that controls K^+^ content in the shoots, named *qSKC1* in a F_2_ and an equivalent F_3_ population derived from a cross between a salt-tolerant cultivar Nona Bokra and a salt-sensitive cultivar Koshihikari (Lin et al. 2004). *qSKC1* was located on chromosome 1 and encoded an ion transporter of the HKT family, which transported excess Na^+^ from the shoots back to the roots and improved the salt tolerance of rice (Lin et al. 2004; Hauser and Horie 2010). Other study identified a QTL that overlaps with *qSKC1* in a F_8_-generation recombinant inbred line (RIL) derived from Pokkali and IR29, *Saltol*, which regulated the K^+^/Na^+^ balance of rice plants under salt stress, and they finally speculated that *Saltol* and *qSKC1* may encode the same gene, *OsHKT1* (Bonilla et al. 2002; Thomson et al. 2010; Niones 2013). Huang et al. (2009) cloned and characterized *DST* (drought and salt tolerance) encoding a zinc finger transcription factor that negatively regulates stomatal closure by directly regulating genes related to H_2_O_2_ homeostasis (Huang et al. 2009). He et al. (2019) fine mapped a *qSE3* related to the rapid germination and seedling establishment of rice seeds under high salt stress. This gene encoded the potassium ion transporter OsHAK21, which promoted the accumulation of ABA during the germination stage of rice seeds (He et al. 2019). On the other hand, some salt tolerance genes were obtained by reverse genetics methods, namely, *SNAC1*, *SNAC2*, *NAP*, *ZFP252*, *ZFP182*, etc. (Hu et al. 2008; Xu et al. 2008; Huang et al. 2012; Chen et al. 2014).

In order to better analyze the genetic mechanism of salt tolerance in rice, a high-generation population of 160 RILs derived from the cross between Luohui 9 (*indica*) and RPY geng (*japonica*) was employed to map the quantitative trait locus (QTLs) for salt tolerance under the salt stress simulated with 125 mM NaCl Yoshida solution, using survival rate as the index. We then determined the functions of the newly identified QTLs through comparing the survival rates of RTLs with different genotypes. RNA-seq analysis of root tissues from Luohui 9 and RPY geng treated with salt stress (100 mM NaCl Yoshida solution) for 3 days and 7 days was performed to identify differential genes (DEGs) within QTLs, which combined with genome annotation to predict candidate genes within QTLs.

## Materials and Methods

### Plant Materials and Treatments

RPY geng, Luohui 9, and their derived high-generation recombinant inbred lines (RILs, F_15_, 160 lines) were used in this study. 14-day-old seedlings of every line were treated with 125 mM NaCl Yoshida solution for 7 days for salt treatment. After salt treatment, the seedlings were cultivated in Yoshida solution for 7 days and the survival rates of the seedlings were calculated. For each RIL, 30 seedlings were performed. All treatments were placed randomly with three replications.

For RNA-seq sequencing, rice seedlings were grown in 96-well PCR plates with Yoshida solution (Coolaber, Beijing, China) replaced every 2 days, 26 °C, and a 16/8 h light/dark photoperiod, 60% relative humidity in plant growth incubators (ZSX1500GS, Jingshen Instrument, Shanghai, China) for 14 days. Fourteen-day-old seedlings of RPY geng and Luohui 9 were changed into 100 mM NaCl Yoshida solution for salt stress treatment. Then, we sampled root tissues at 3-day (3d), and 7-day (7d), respectively. Three independent biological replicates were prepared for each treatment and at least 30 seedlings with uniform growth were sampled for each replicate. All samples were collected and immediately stored in liquid nitrogen for the next step of RNA extraction. In addition, we sampled root tissues on 0 days (control), 3 days, and 7 days after salt stress treatment to observe gene expression changes of candidate genes for *qST-5.1*, *qST-6.1*, and *qST-6.2* under salt stress.

### QTL Analysis

The genetic linkage map including 4578 bin blocks (the total bin-map distance was 2356.41 cM) of this RIL population have been previously constructed in our lab (Unpublished). The QTL mapping of survival rates was analyzed by R/qtl (Arends et al. 2010). The CIM interval mapping method was adopted, and the LOD threshold was set at 3.0. The confidence interval was calculated with the function ‘lodint’ (Dupuis and Siegmund 1999), and the drop value was set at 1.5. The genes in QTLs were identified based on the MSU v7.0 (from the Rice Genome Annotation Project, http://rice.plantbiology.msu.edu/index.shtml) and putative functions annotations of genes were also obtained from “Genome Annotation Batch Download” in the Rice Genome Annotation Project website (Kawahara et al. 2013).

### RTM-GWAS Analysis

The vcf file composed of 4578 bins and the survival rate data of 160 RILs was used as the two input files of RTM-GWAS with operating parameters: significant level of 0.01, pre-selection threshold of 0.05, maximum model r-square of 0.95, and others defaults (He et al. 2017). The GWAS analysis results were visualized by CMplot (https://cran.r-project.org/web/packages/CMplot/). In this study, the region of 0.15 Mb upstream and downstream of the significant bin block/marker was regarded as the QTL interval.

### Meta-QTLs Comparison and Known Genes Homology Analysis

41 Meta-QTLs related to salt tolerance were collected from Mansuri et al. study (Mansuri et al. 2020) and the genomic positions of these Meta-QTLs were obtained based on the method we previously described (Kong et al. 2020). Based on location information, Meta-QTLs and identified QTLs were visualized using TBtools (Additional file 2: Table S1) (Chen et al. 2020). On the other hand, 132 salt stress-related known genes involving developmental adjustment, hormonal regulation, ionic homeostasis, nutrient imbalance, osmotic adjustment, ROS scavenging, and salt stress signaling, were collected (Additional file 3: Table S2) (Liu et al. 2021). These known genes further were mapped into Meta-QTLs and our QTLs depending on gene position, respectively. In addition, we identified the homologous genes of the known genes in our QTLs by searching with blastP (Evalue of 1e-20). Of them, *qST-4.1*, *qST-4.2*, and *qST-8.1* were overlapped with *MQTL-4.3*, *MQTL-4.4*, and *MQTL-8.4*, respectively.

### RNA-seq Analysis

RNA extraction, cDNA library construction, and library sequencing on Illumina HiSeq2500 platform of 12 samples were strictly implemented by Biomarker Technologies (Beijing, China) in accordance with standard procedures (Kong et al. 2020). Raw data were filtered by fastp (Chen et al. 2018) and mapped to the Nipponbare genome (MSU v7.0) using hisat2 (Kim et al. 2015) with default parameters. The mapped reads were counted by featureCounts (Liao et al. 2014) and differentially expressed genes (DEGs) in QTLs were identified by DEseq2 with |log_2_^fold change^|≥ 1 and a False Discovery Rate (FDR) < 0.01 (Kong et al. 2020). The heatmap of DEGs was also conducted by TBtools (Chen et al. 2020).

Finally, three randomly selected candidate DEGs in QTLs were verified by RT-PCR according to the previously described method (Kong et al. 2019). All primers of RT-PCR were designed by Primer 5.0 software (Additional file 4: Table S3). The qRT-PCR reaction (10 μL) was formulated using the 2 X SYBR Green qPCR Master Mix (US Everbright®Inc., Suzhou, China). All qRT-PCRs were carried out on a CFX96 Touch™ Real-Time PCR Detection System (Bio-Rad, Hercules, CA, USA). The gene expression fold change was calculated by the 2^−ΔΔCT^ method from three biological replicates.

## Results

### Salt Tolerance Performance of Parents and RILs

We found that RPY geng had a higher survival rate than Luohui 9 (81% vs 31%) (Additional file 1: Figure S1). The RILs showed differentiation of survival rate from 0 to 100% and 16 RILs exhibited high salt stress tolerance with 100% survival rate. These results indicated that *japonica* rice is more tolerant to salt stress than *indica* rice, and *indica*-*japonica* hybridization is a feasible strategy to breed new rice varieties that are tolerant to high salt stress.

### QTL Mapping Result of Survival Rate

**Fig. 1:**
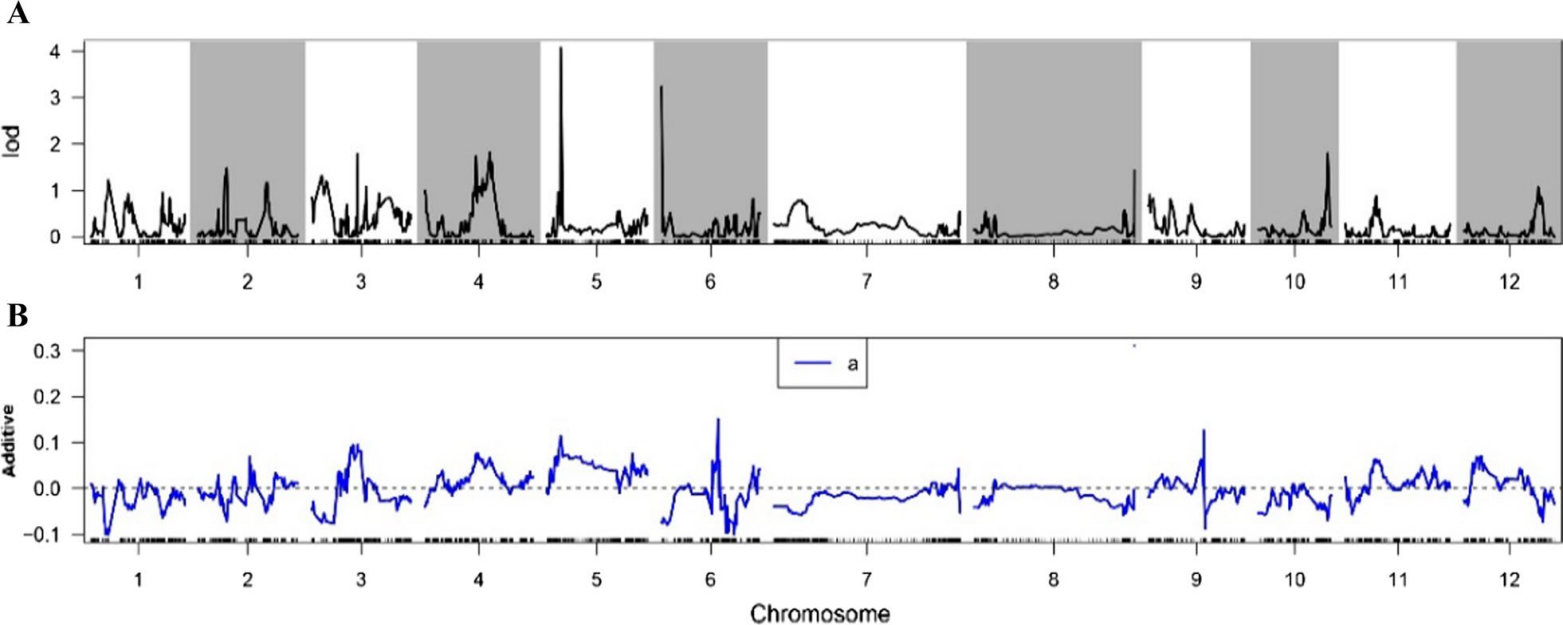
QTL mapping result of survival rate from R/qtl. **A** Lod values of QTLs; **B** additive values of QTLs

**Fig. 2:**
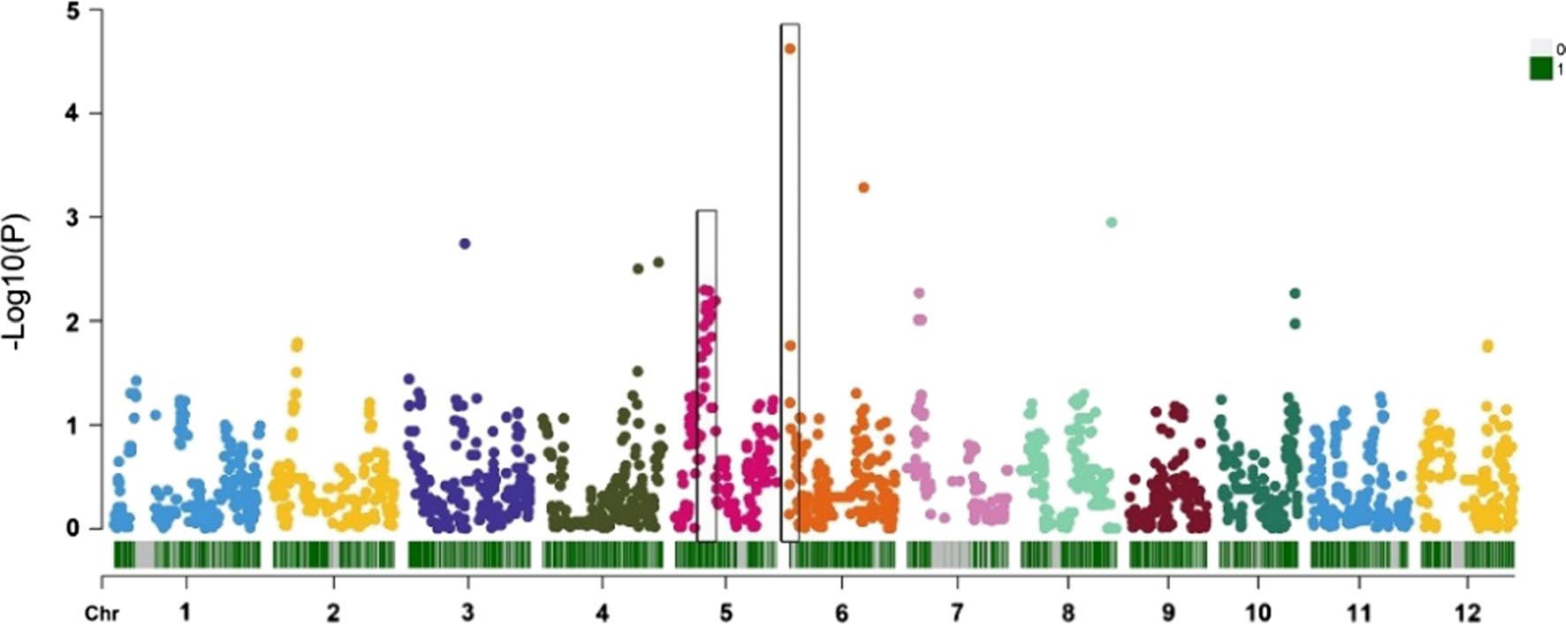
QTL mapping result of survival rate from RTM-GWAS. The black box represents the QTL repeatedly detected by R/qtl and RTM-GWAS

The QTL mapping of R/qtl identified two survival rate-related QTLs distributed on chromosome 5 and chromosome 6 with phenotypic variance explained of 11.05 and 8.90 and additive effect of 0.11 and − 0.07, respectively (Fig. 1 and Additional file 5: Table S4). On the other hand, seven quantitative trait bin blocks (QTBs) were identified by RTM-GWAS and the regions of 0.15 Mb upstream and downstream of seven QTBs were regarded as the confidence interval of QTLs (Table 1 and Fig. 2). Of them, *qST-5.1* and *qST-6.1* were redefined using the intersection result of RTM-GWAS and R/qtl’s QTL position (Table 1 and Additional file 5: Table S4).

**Table 1:** Details of QTLs and QTBs from RTM-GWAS

QTL	Chr	Size of QTL	Pos of QTL	QTB	Pos of QTB	− logP	PEV (%)
*qST-3.1*	Chr3	0.31	16,994,792–17,308,890	Block107986	17,144,792–17,158,890	2.95	4.8706984
*qST-4.1*	Chr4	0.32	28,073,408–28,389,701	Block134443	28,223,408–28,239,701	2.74	4.12779992
*qST-4.2*	Chr4	0.31	34,076,941–34,389,322	Block138596	34,226,941–34,239,322	2.56	5.27897414
*qST-5.1*	Chr5	0.20	8,337,466–8,540,272	Block142677	8,487,466–8,513,778	2.29	4.16431194
*qST-6.1*	Chr6	0.04	158,939–203,553	Block151076	308,939–342,522	4.62	12.6821866
*qST-6.2*	Chr6	0.34	21,811,442–22,148,865	Block159231	21,961,442–21,998,865	3.28	8.1133099
*qST-8.1*	Chr8	0.31	26,804,808–27,118,145	Block190194	26,954,808–26,968,145	2.50	4.44373423

The QTL confidence interval was from the 0.15 Mb upstream and downstream of QTB. *qST-5.1* and *qST-6.1* were redefined using the intersection result of RTM-GWAS and R/qtl’s QTL position. Chr, Pos, QTB, and PEV were the abbreviations of chromosome, position, quantitative trait blocks, and position effect variegation, respectively

### Identification of Novel QTLs Related to Salt Tolerance

**Fig. 3:**
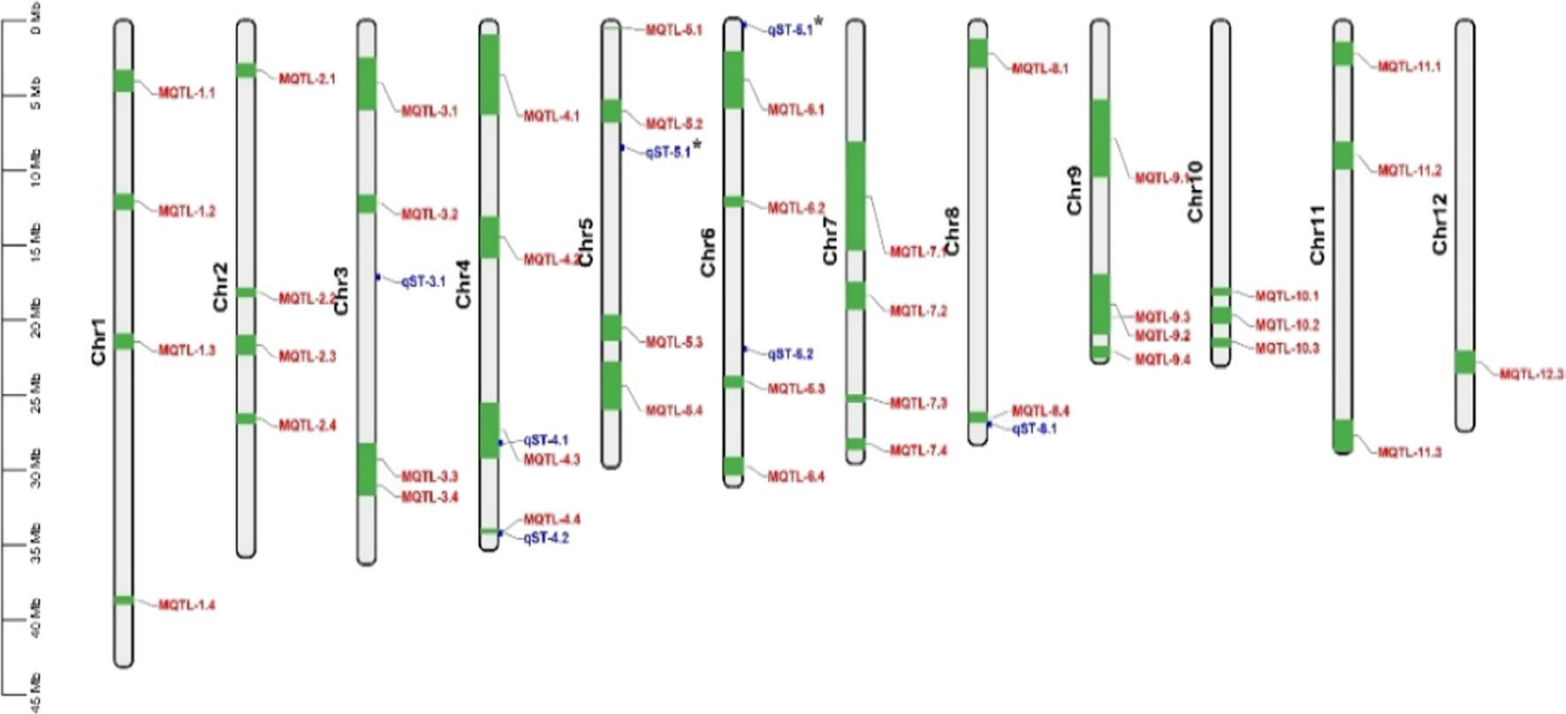
The positions of our QTLs and Meta-QTLs. Red and blue QTL represents Meta-QTLs and our QTLs. *Note* The green and blue boxes represent the Meta-QTLs and our QTLs intervals, respectively

In order to analyze whether the QTLs we identified are new salt-stressed QTLs, we made a comparative analysis between our QTLs and Meta-QTLs. As shown in Fig. 3, *qST-4.1*, *qST-4.2*, and *qST-8.1* were completely/partially overlapped with *MQTL-4.3*, *MQTL-4.4*, and *MQTL-8.4*, respectively. Comparative analysis between our QTLs and Meta-QTLs implied that *qST-3.1*, *qST-5.1*, *qST-6.1*, and *qST-6.2* were new salt stress tolerance QTLs.

### Function Confirmation of the Four Novel QTLs for Seedling Survival Rate

**Fig. 4:**
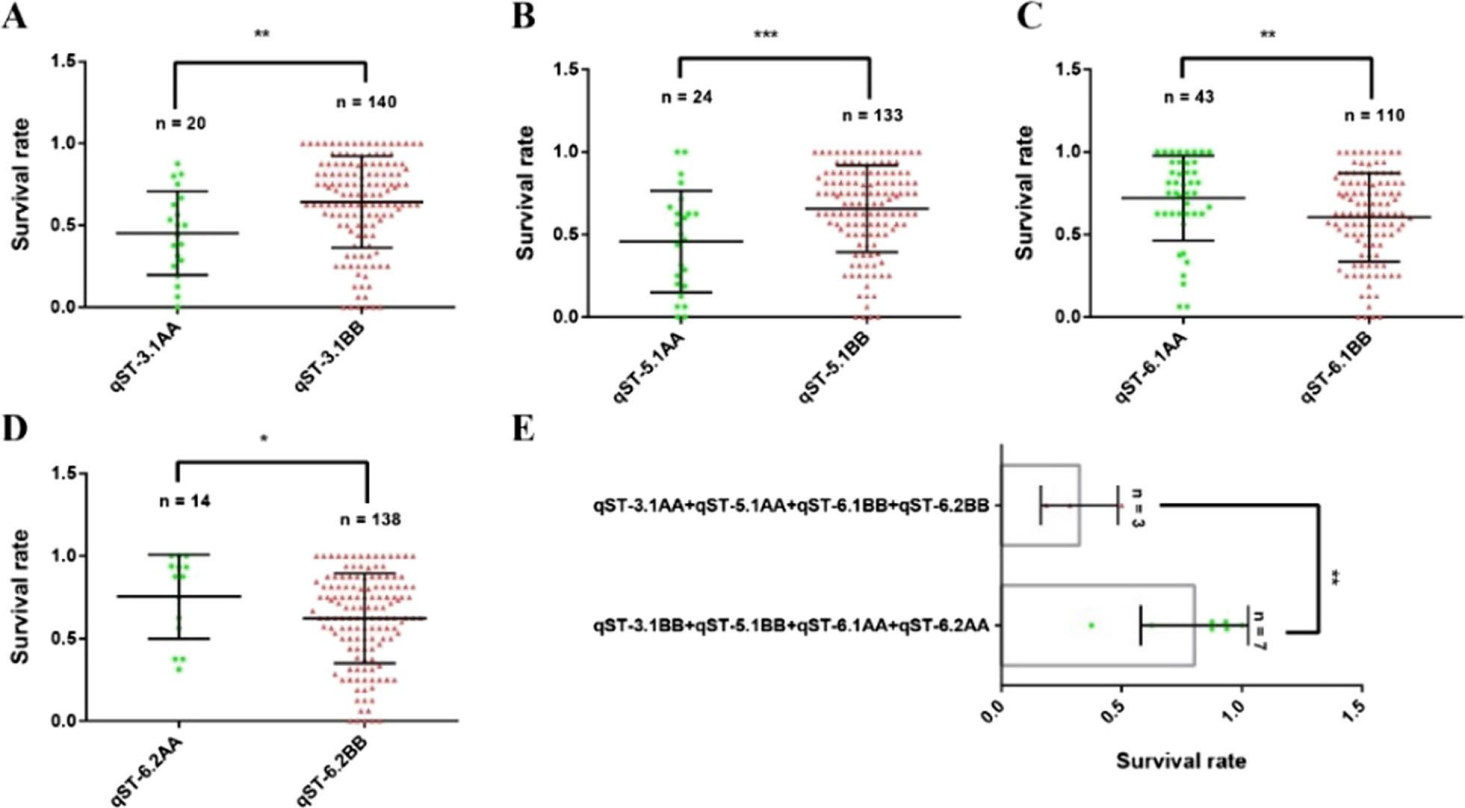
The survival rate of rice seedlings with different genotyping of RILs. A. *qST-3.1*; B. *qST-5.1*; C. *qST-6.1*; D. *qST-6.2*; E. Aggregation of *qST-3.1*, *qST-5.1*, *qST-6.1*, and *qST-6.2*. *Note* AA means RPY geng and BB means Luohui 9

We next wondered whether these four new QTLs (*qST-3.1*, *qST-5.1*, *qST-6.1*, and *qST-6.2*) have any effect on the rice seedling survival rate. We therefore divided all RILs into RPY geng (AA) and Luohui 9 (BB) genotype RILs and compared the survival rates of these two genotype RILs. Luohui 9 genotyping RILs showed higher survival rates than RPY geng genotyping RILs in *qST-3.1* and *qST-5.1* (Fig. 4A, B). Interestingly, *qST-6.1*, and *qST-6.2* showed the opposite result relative to *qST-3.1* and *qST-5.1* (Fig. 4C, D). This situation implied that the synergistic genotypes of these four QTLs are not the same, and the aggregation of the synergistic genotypes of these four QTLs may promote the survival rate of rice seedlings. As expected, RILs aggregated by synergistic genotypes showed a higher survival rate compared to RILs aggregated by non-synergistic genotypes (Fig. 4E). These results not only indicated that the four novel QTLs we identified had regulatory effects on the survival rate of seedlings but also emphasized that their synergistic aggregation can enhance the survival rate of seedlings.

### Mapping Known Genes into Meta-QTLs and our QTLs

**Fig. 5:**
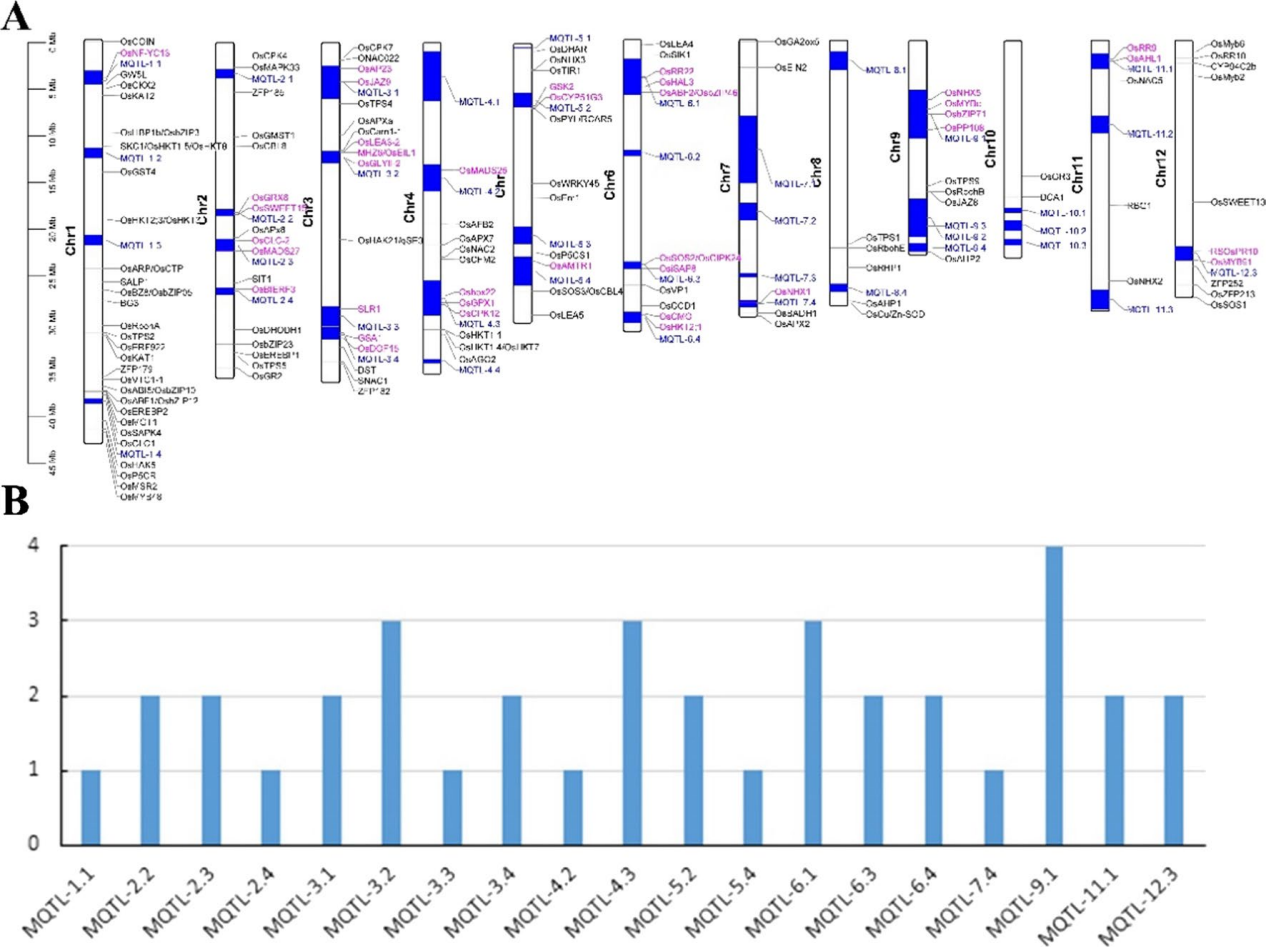
Known genes into Meta-QTLs. **A** Distribution of known genes and Meta-QTLs on 12 chromosomes; **B** The number of known genes in Meta-QTLs. *Note* Blue represents Meta-QTLs, and red represents genes that fall into Meta-QTLs in **A**

**Fig. 6:**
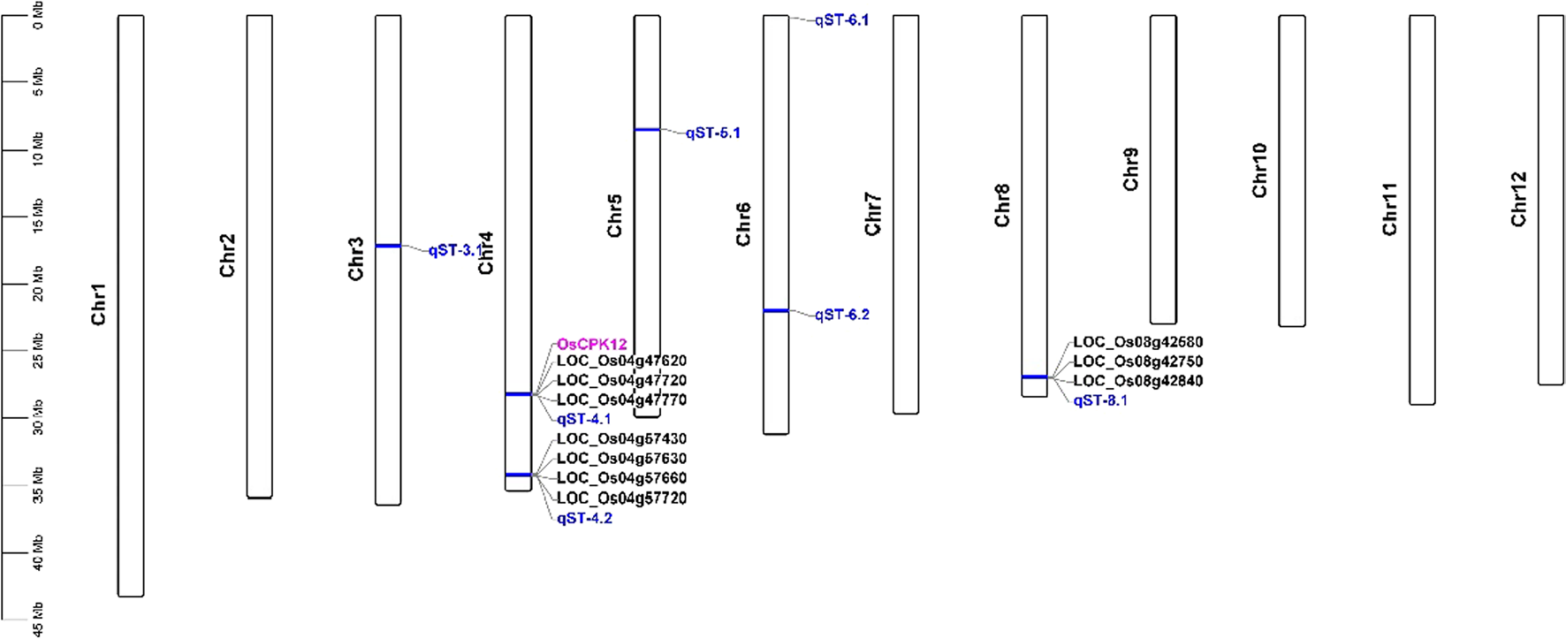
Known genes and their homologous genes in our QTLs. *Note* Red represents known genes that fall into QTLs, and black represents homologous genes of known genes in QTLs

We subsequently assessed the known genes of Meta-QTLs and our QTLs to preliminarily determine the candidate genes in QTLs. Of 41 Meta-QTLs, 19 contained a total of 37 known salt stress tolerance genes among chromosome 1, 2, 3, 4, 5, 6, 7, 9, 11, and 12 (Fig. 5A). Several Meta-QTLs contained more than one known gene, such as *MQTL-9.1* had four known genes (Fig. 5B and Additional file 6: Table S5). Similarly, one known gene (*OsCPK12*) was found in *qST-4.1* (Fig. 6). In addition, we identified three, four, and three homologous genes of known genes in *qST-4.1*, *qST-4.2*, and *qST-8.1* (Fig. 6 and Additional file 7: Table S6). However, no known genes or homologous genes of known genes were found in the four newly discovered QTLs (*qST-3.1*, *qST-5.1*, *qST-6.1*, and *qST-6.2*).

### DEGs in our QTLs

**Fig. 7:**
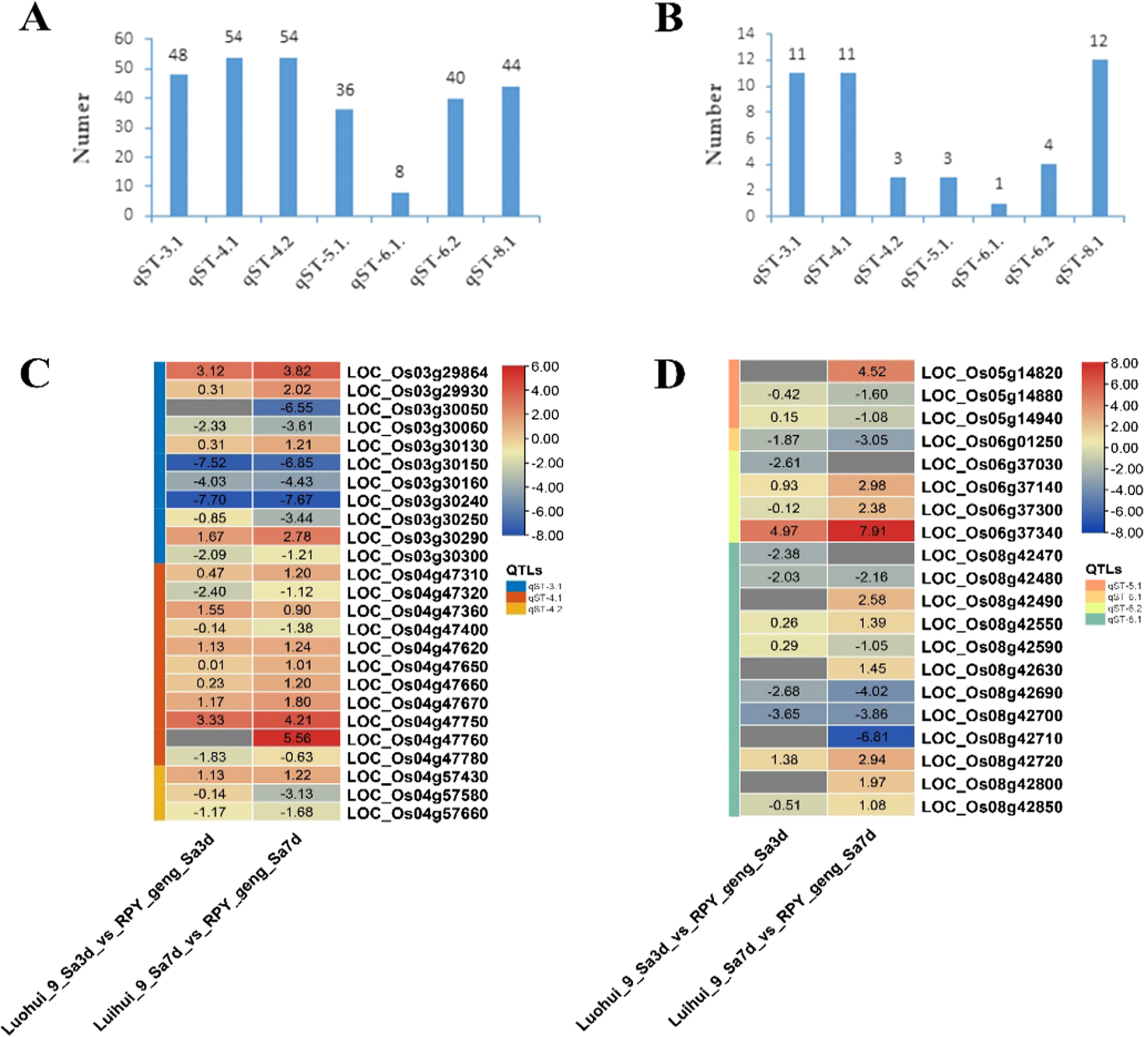
DEGs analysis in QTLs. **A** Statistics of the number of genes in QTLs; **B** Statistics of the number of DEGs in QTLs; **C**, **D** Heat map of DEGs in QTLs. *Note* Sa3d and Sa7d mean salt treatment (100 mM NaCl Yoshida solution) for 3 days and for 7 days, respectively

We discovered that seven QTLs contained a total of 284 genes, of which there were only 8 genes in *qST-6.1* (Fig. 7A and Additional file 8: Table S7). Then, we obtained DEGs of QTLs between Luohui 9 and RPY geng to predict the candidate genes of QTLs. There were varying amounts of DEGs in these 7 QTLs, from 1 to 12 (Fig. 7B–D). For example, *LOC_Os03g30150*, *LOC_Os03g30160*, and *LOC_Os03g30240* showed a higher expression level in RPY geng than that in Luohui 9 (Fig. 7C). Notably, *qST-6.1* contained only one DEG (*LOC_Os06g01250*) with a higher expression level in RPY geng than that in Luohui 9, which may play an important role in salt-tolerant species (Fig. 7D). Similarly, *LOC_Os06g37300* was the only DEG in *qST-6.2*. *qST-5.1* contained only two DEGs, *LOC_Os05g14820* and *LOC_Os05g14880*. We accepted *LOC_Os05g14880* as the candidate gene for *qST-5.1* based on gene annotation, because *LOC_Os05g14880* encodes a stress-related proline-rich protein (Additional file 9: Table S8). Interestingly, the two DEGs (*LOC_Os04g57430* and *LOC_Os04g57660*) encoding receptor-like kinases in *qST-4.2* were homologous genes of the known salt tolerance-related gene (Additional file 7: Table S6, Additional file 9: Table S8), *OsSIK1,* which supports these two genes as candidate genes for *qST-4.2*. The candidate genes of *qST-3.1*, *qST-4.1*, and *qST-8.1* need to be further explored due to the inclusion of too many DEGs (> 7). It was worth mentioning that *LOC_Os04g47620* was a homologous gene of *OsSIK1* and *SIT1* genes and showed differential expression level between RPY geng and Luohui 9 under salt stress (Additional file 7: Table S6, Additional file 9: Table S8), which suggested that *LOC_Os04g47620* was very likely to be a candidate gene for *qST-4.1*. In summary, the integration of QTL mapping, RNA-seq analysis, and gene annotation provided credible candidate genes for the identified QTLs, which was very important for subsequent map-based cloning and gene function verification.

Finally, three genes (*LOC_Os03g30250*, *LOC_Os03g30290*, and *LOC_Os08g42720*) were randomly selected for RT-PCR analysis to verify the accuracy of RNA-seq, and the RT-PCR results were consistent with the RNA-seq results (Additional file 1: Figure S2).

### Expression Characterization, Sequence Comparison, Haplotype Analysis of Candidate Genes for qST-5.1, qST-6.1, and qST-6.2

**Fig. 8:**
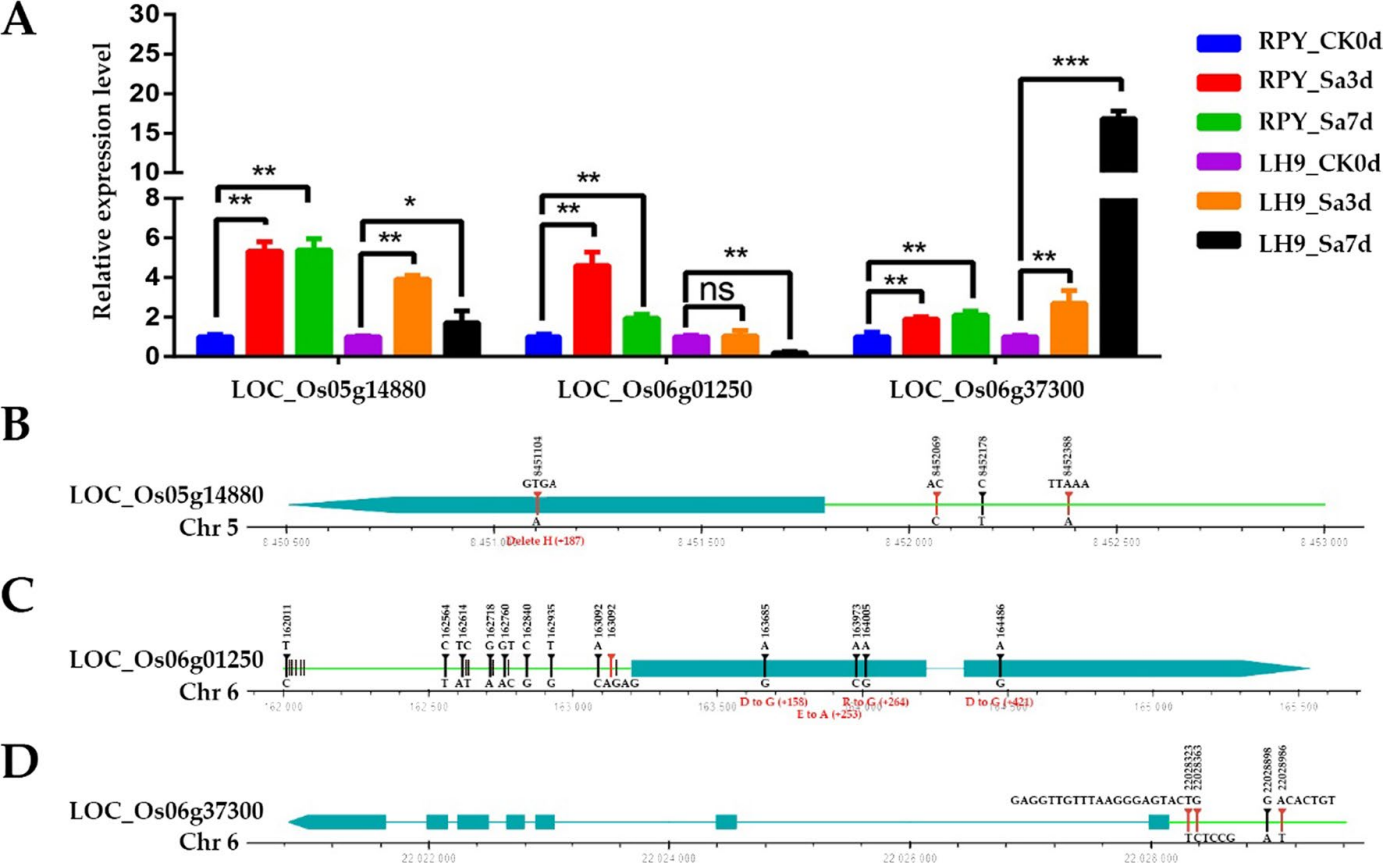
Gene expression changes and sequence alignments of *LOC_Os05g14880*, *LOC_Os06g01250*, and *LOC_Os06g37300* between RPY geng and Luohui 9. **A** Changes in gene expression levels in the roots of RPY geng and Luohui 9 after 3 and 7 days of salt stress treatment (100 mM NaCl Yoshida solution for salt stress treatment); **B** Sequence alignment result of *LOC_Os05g14880*; **C** Sequence alignment result of *LOC_Os06g01250*; **D** Sequence alignment result of *LOC_Os06g37300*. *Note* RPY means RPY geng; LH9 means Luohui 9; CK0d means control (0 day); Sa3d and Sa7d mean salt treatment for 3 days and for 7 days, respectively. The upper mutations belong to RPY geng and the lower mutations belong to Luohui 9 in **B**–**D**

To preliminarily explore the functions of candidate genes for *qST-5.1*, *qST-6.1*, and *qST-6.2*, we analyzed the expression profiles, sequence differences, and haplotypes of *LOC_Os05g14880*, *LOC_Os06g01250*, and *LOC_Os06g37300* in rice populations. The results of qRT-PCR showed that these three genes showed different expression levels in the roots of RPY geng and Luohui 9 under salt stress. *LOC_Os05g14880* showed a higher level of up-regulation in RPY geng than that of Luohui 9 (Fig. 8A). The expression level of *LOC_Os06g01250* were significantly up-regulated in RPY geng on 3 days and 7 days after salt treatment, while *LOC_Os06g01250* had a significant down-regulation in Luohui 9 on 7 days after salt treatment. Differently, *LOC_Os06g37300* showed a higher level of up-regulated expression fold in Luohui 9 than that of RPY geng. In addition, the tissue expression profiles of these three genes were further characterized from MBKbase (http://www.mbkbase.org/rice) (Additional file 1: Figure S3). *LOC_Os05g14880* showed superior expression in root-tip and shoot than other organizations. *LOC_Os06g01250* has a higher level of expression in shoot, stem, and leaf relative to other tissues. *LOC_Os06g37300* was the advantage expressed in leaf and panicle.

The alignment results of *LOC_Os05g14880*, *LOC_Os06g01250*, and *LOC_Os06g37300* sequences revealed that RPY geng and Luohui 9 have multiple types of mutations in the 1.2-kilobase (Kb) promoter, coding sequence (CDS) (Fig. 8B–D). For example, one indel in CDS as well as two indels and one snp were found in *LOC_Os05g14880* (Fig. 8B). Four non-synonymous snps were detected in CDS of *LOC_Os06g01250* and 18 snp and one indel were found in 1.2 Kb promoter of *LOC_Os06g01250* (Fig. 8C). Notably, there was no sequence variation between RPY geng and Luohui 9 in the CDS of *LOC_Os06g37300*, but three long-indels (> 5 bp) and one snp in the 1.2 Kb promoter (Fig. 8D). These four variations can be overlapped by the results of rice population variation detection (Additional file 1: Figure S4). In view of the significant difference in gene expression levels of these three genes in RPY geng and Luohui 9, we speculated that the variation in the promoter region, especially the transcription factor binding region, is the cause of the differential expression of these genes.

Population level variation sites in these three sequences, which covers the 1.2-Kb, CDS, and 3’ untranslated region (UTR), were obtain from the rice genome variation database (Rice Variation Map v2.0, http://ricevarmap.ncpgr.cn/) of 4,726 rice accessions. *LOC_Os05g14880*, *LOC_Os06g01250*, and *LOC_Os06g37300* contained 150 (130 snps and 20 indels), 104 (94 snps and 10 indels), and 490 (378 snps and 112 indels) variation sites, respectively (Additional file 1: Figure S4A, S4C, S4E). Here, 28, 24, 29 variation sites in UTR and CDS of *LOC_Os05g14880*, *LOC_Os06g01250*, and *LOC_Os06g37300* were used for haplotype analysis (Additional file 10: Table S9, Additional file 11: Table S10, Additional file 12: Table S11). *LOC_Os05g14880* totally contained 12 haplotypes, which were named haplotypes I – XII (hap I – hap XII). Of them, Hap I was present mainly in *japonica* cultivars and Hap II mainly in *indica* cultivars (Additional file 1: Figure S4B and Additional file 10: Table S9). *LOC_Os06g01250* had eight haplotypes, Hap I, Hap VI, and Hap VII were present mainly in *japonica* cultivars and Hap II, Hap III, Hap IV, and Hap V mainly in *indica* cultivars (Additional file 1: Figure S4D and Additional file 11: Table S10). *LOC_Os06g37300* harbored 11 haplotypes, Hap III was the main haplotype including 2573 rice cultivars, namely, 1915 *indica*, 618 *japonica*, and 40 *intermediate* cultivars (Additional file 1: Figure S4F and Additional file 12: Table S11).

In summary, gene expression differences and gene sequence variations between RPY geng and Luohui 9 suggested that *LOC_Os05g14880*, *LOC_Os06g01250*, and *LOC_Os06g37300* can be accepted as candidate genes for *qST-5.1*, *qST-6.1*, and *qST-6.2*. These results suggested that these genes have a potentially important role in salt stress tolerance, which provides confident targets for future validation through Crispr-Cas9 and overexpression methods.

## Discussion

There are significant differences in the salt tolerance of rice at various growth stages. The seedling stage and young panicle differentiation stage are more sensitive to salt stress, while the bud stage, the tillering stage, and maturity stage are relatively strong in salt tolerance (Khan and Abdullah 2003). The survival rate under salt stress is a comprehensive performance of salt tolerance mechanisms, a reliable indicator to measure the strength of rice salt tolerance, and it is simple and convenient to observe (Liang et al. 2015). In this study, 2-week-old rice seedlings of Luohui 9, RPY geng, and RILs were selected for salt treatment. The difference in salt stress tolerance between Luohui 9 and RPY geng supported the previous studies that *japonica* rice is more tolerant to salt stress than *indica* rice (Islam et al. 2019; Kong et al. 2019). We speculated that this difference in salt tolerance between these subspecies may be due to the loss of more abiotic stress-related tolerance genes in *indica* than in *japonica* during the subspecies independent evolution, which was supported by our comparative family gene analysis between Luohui 9 and RPY geng (Unpublished data). Here, seven salt tolerance QTLs related to the seedling survival rate were identified. Compared with previous Meta-QTLs, four QTLs were previously unreported and were functionally validated by RILs (Fig. 4), which provided novel targets for rice salt stress tolerance breeding. On the other hand, our experiments demonstrated the feasibility of *indica*-*japonica* hybridization to breed stronger salt tolerant varieties and successfully selected several high salt-tolerant RILs. Our RILs genotyping results from four novel QTLs (Fig. 4) showed that the excellent performance in salt tolerance of high salt-tolerant RILs can be derived from synergistic genotype aggregation of the parental QTLs. These results emphasized that the QTLs we have identified are of great significance for improving the tolerance of rice to salt stress.

To analyze the QTLs of rice salt stress tolerance, this study adopted two analysis methods, namely, linkage mapping and RTM-GWAS. We found that RTM-GWAS was effective in identifying QTLs in a bi-parental RILs population and identified more QTLs that overlapped with previous Meta-QTLs than that in linkage mapping. Similarly, RTM-GWAS showed a more effective QTL detection rate in soybean bi-parental RILs populations, and lower false positives compared to linkage mapping or traditional GWAS (Pan et al. 2018; Khan et al. 2020; Chang et al. 2021; Zou et al. 2021). Our result proved that integration of linkage mapping and RTM-GWAS can highlight important QTLs, such as *qST-5.1* and *qST-6.1* in this study.

In the past 20 years, a large number of QTLs have been mapped in different rice populations by various methods, such as GWAS, QTL-seq, or BSA-seq, but only a little genes have been cloned and most candidate genes were uncertain due to the large QTL interval (Thomson et al. 2010; Kong et al. 2020). DEGs mapping in QTLs or Meta-QTLs can effectively highlight candidate genes (Yang et al. 2019; Kong et al. 2020). For example, Lei et al. (2020) reported that *OsSAP16* was the candidate gene of *qRSL7* under salt stress using QTL-Seq and RNA-Seq (Lei et al. 2020). In this study, we treated RPY geng and Luohui 9 with salt stress and performed RNA-seq analysis on their roots. We found that the number of DEGs in QTLs was extremely small, which greatly reduced the numbers of candidate genes, especially *qST-6.1*. Only one DEG (*LOC_Os06g01250*) was found in *qST-6.1* or *qST-6.2*, encoding cytochrome P450 protein. Magwanga et al. (2019) reported that two cytochrome P450 genes (*Gh_D07G1197* and *Gh_A13G2057*) played crucial roles in enhancing drought and salt stress tolerance in *Gossypium hirsutum* (Magwanga et al. 2019). *CYP709B3*, a cytochrome P450 monooxygenase gene involved in salt tolerance in *Arabidopsis* (Mao et al. 2013). In addition, a cytochrome P450, *OsDSS1*, was involved in growth and drought stress responses in rice (*Oryza sativa* L.) (Tamiru et al. 2015). These results proved that cytochrome P450s were important regulators of abiotic responses. We thus speculated that *LOC_Os06g01250* was the candidate gene in *qST-6.1*, which was supported by DEG and previous studies. In addition, *qST-5.1* only contained two genes, namely, *LOC_Os05g14820* and *LOC_Os05g14880*. In *Nicotiana tabacum*, *NtProRP1* encoding a novel proline-rich protein was an important osmotic stress-responsive factor and had specifically functions in pollen tube growth and early embryogenesis (Chen et al. 2013). An *Arabidopsis* zinc-finger gene, *MEDIATOR OF ABA-REGULATED DORMANCY 1* (*MARD1*), with a proline-rich domain mediated ABA-regulated seed dormancy in *Arabidopsis* (He and Gan 2004). These results indicated that proline-rich proteins were stress-responsive factors involving phytohormone-related pathways. What’s more, *qST-4.2*, *qST-4.1*, *qST-3.1*, and *qST-8.1* contained only 2, 7, 10, and 12 candidate genes, respectively, which greatly reduced the workload of later experimental functional verification.

## Conclusion

In the present study, the salt stress tolerance differences of Luohui9, RPY geng, and their derived high-generation RILs were observed. We have successfully bred several high salt tolerance RILs through the hybrid breeding strategy of *indica* x *japonica* rice. Integrating linkage mapping, RTM-GWAS, Meta-QTLs comparison results identified of seven salt stress tolerance QTLs, including four new QTLs with extremely small physical intervals. The survival rate results of RILs demonstrate the functional effectiveness of the four new QTLs. The comparative transcriptome analysis of RPY geng and Luohui 9 highlighted the candidate genes in QTLs, namely, *LOC_Os06g01250* in *qST-6.1*, *LOC_Os06g37300* in *qST-6.2*, *LOC_Os05g14880* in *qST-5.1*. Several randomly selected candidate genes can be further improved by qRT-PCR verification. Our results provided a new source for cloning of genes associated with salt resistance and molecular breeding in rice.

## Supplementary Information

**Additional file 1**. **Figure S1**. The survival rate of rice seedlings (Luohui 9, RPY geng, and RILs) after 7 days of salt stress treatment and 7 days of recovery; **Figure S2**. Three randomly selected candidate genes were verified by RT-PCR. **Figure S3**. Expression levels of *LOC_Os05g14880*, *LOC_Os06g01250*, and *LOC_Os06g37300* in rice tissues from http://www.mbkbase.org/rice. **Figure S4**. Variant sites and haplotype results of *LOC_Os05g14880*, *LOC_Os06g01250*, and *LOC_Os06g37300* from the rice genome variation database of 4,726 rice accessions (Rice Variation Map v2.0, http://ricevarmap.ncpgr.cn/).

**Additional file 2**. **Table S1**. Meta-QTLs used in this study.

**Additional file 3**. **Table S2**. Known genes used in this study.

**Additional file 4**. **Table S3**. Primers of DEGs were used in this study.

**Additional file 5**. **Table S4**. QTLs from R/qtl analysis.

**Additional file 6**. **Table S5**. Known genes in Meta-QTLs.

**Additional file 7**. **Table S6**. Known genes and their homologous genes in our identified QTLs

**Additional file 8**. **Table S7**. Genes in our QTLs.

**Additional file 9**. **Table S8**. Candidate genes in our QTLs.

**Additional file 10**. **Table S9**. Haplotype analysis result of *LOC_Os05g14880*.

**Additional file 11**. **Table S10**. Haplotype analysis result of *LOC_Os06g01250*.

**Additional file 12**. **Table S11**. haplotype analysis result of *LOC_Os06g37300*.

## Data Availability

The RNA-seq data used in the study are publicly available from the NCBI (BioProject ID PRJNA732136).

## Abbreviations

GWAS: Genome-wide association study
ST: Salt tolerance
DEGs: Differentially expressed genes
QTLs: Quantitative trait locus
ROS: Reactive oxygen species
RILs: Recombinant inbred lines
FDR: False discovery rate
QTBs: Quantitative trait bin blocks
