# Supplementary material for: Uncovering the Novel QTLs and Candidate Genes of Salt Tolerance in Rice with Linkage Mapping, RTM-GWAS, and RNA-seq
Source: Rice (N Y). 2021 Nov 14;14:93. doi: 10.1186/s12284-021-00535-3 (PMC8590990; doi:10.1186/s12284-021-00535-3)
Supplement: Supplementary file 1 — Additional file 1. Figure S1. The survival rate of rice seedlings (Luohui 9, RPY geng, and RILs) after 7 days of salt stress treatment and 7 days of recovery; Figure S2. Three randomly selected candidate genes were verified by RT-PCR. Figure S3. Expression levels of LOC_Os05g14880, LOC_Os06g01250, and LOC_Os06g37300 in rice tissues from http://www.mbkbase.org/rice. Figure S4. Variant sites and haplotype results of LOC_Os05g14880, LOC_Os06g01250, and LOC_Os06g37300 from the rice genome variation database of 4,726 rice accessions (Rice Variation Map v2.0, http://ricevarmap.ncpgr.cn/). [file 12284_2021_535_MOESM1_ESM.docx]

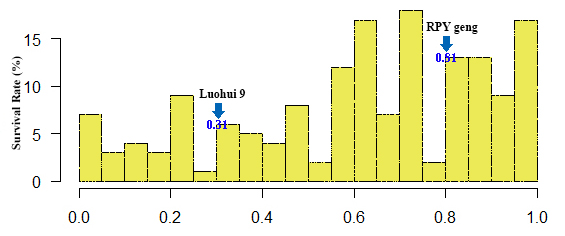


**Figure S1.** The survival rate of rice seedlings (Luohui 9, RPY geng, and RILs) after 7 days of salt stress treatment and 7 days of recovery.


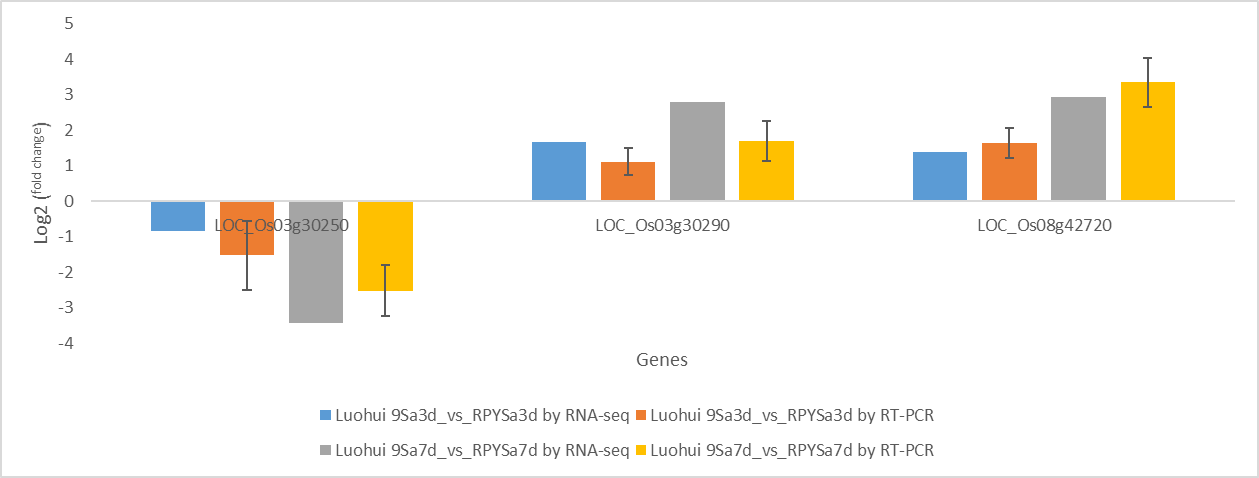


**Figure S2.** Three randomly selected candidate genes were verified by RT-PCR.


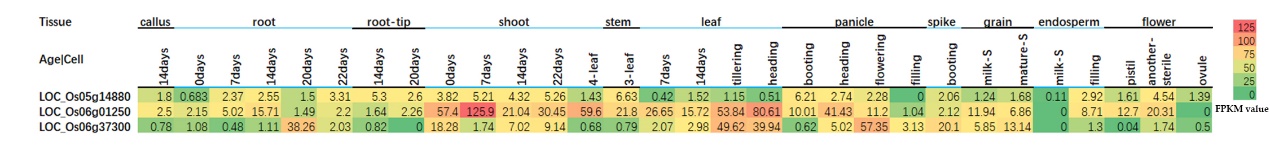


**Figure S3.** Expression levels of *LOC_Os05g14880*, *LOC_Os06g01250*, and *LOC_Os06g37300* in rice tissues from <http://www.mbkbase.org/rice>.


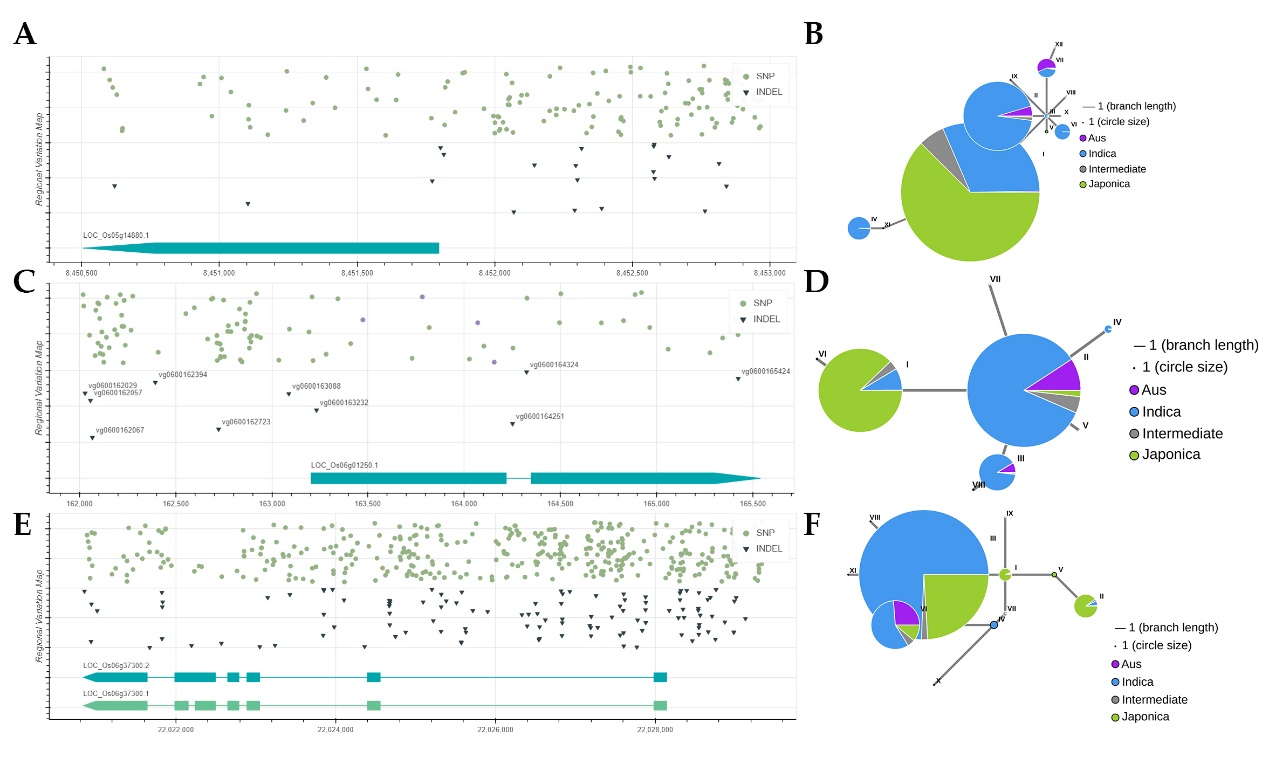


**Figure S4.** Variant sites and haplotype results of *LOC_Os05g14880*, *LOC_Os06g01250*, and *LOC_Os06g37300* from the rice genome variation database of 4,726 rice accessions (Rice Variation Map v2.0, http://ricevarmap.ncpgr.cn/).
